# GII.23/24/25 noroviruses recognize glycans via a conventional glycan-binding site

**DOI:** 10.3389/fmicb.2026.1767002

**Published:** 2026-03-03

**Authors:** Hanbo Li, Xin Cong, Xiaoman Sun, Jianxun Qi, Xinyu Li, Miao Jin, Zhaojun Duan

**Affiliations:** 1 Guangdong Provincial Key Laboratory of Agro-Animal Genomics and Molecular Breeding, College of Animal Science, South China Agricultural University, Guangzhou, China; 2 National Institute for Viral Disease Control and Prevention, China CDC, Beijing, China; 3 Department of Infectious Disease, Children’s Hospital, National Clinical Research Center for Child Health, Zhejiang University School of Medicine, Hangzhou, China; 4 Institute of Microbiology, Chinese Academy of Sciences, Beijing, China; 5 College of Basic Medicine, Chengde Medical University, Chengde, China

**Keywords:** GII.23/24/25 P domains, H disaccharide, histo-blood group antigens, noroviruses, structures

## Abstract

**Introduction:**

Human noroviruses (HuNoVs) are genetically diverse RNA viruses that cause acute gastroenteritis, with genogroup II (GII) accounting for over 90% of global infections. Glycans, particularly histo-blood group antigens (HBGAs), have been identified as attachment factors or receptors for HuNoVs infection. However, the glycan-binding receptors of the later-identified GII genotypes GII.23/24/25 remain elusive.

**Methods:**

We used saliva- and glycan-based ELISA assays to identify the binding spectra of GII.23/24/25 strains. We also solved the crystal structures of their P domains, including the GII.25 P domain in complex with the H disaccharide. Single-point mutagenesis was performed to identify key residues involved in glycan binding.

**Results:**

The P domains of GII.24 and GII.25 can recognize multiple types of saliva samples, including both A/B/O secretor and nonsecretor individuals. In contrast, GII.23 primarily binds to B secretor saliva samples. Furthermore, GII.23/24 P domains are able to interact with the H disaccharide, whereas GII.25 exhibits binding affinity for both H disaccharide and B trisaccharide. Crystal structures of GII.23/24/25 P domains revealed high structural similarity, and the complex of GII.25 P domains with H disaccharide was resolved. Single-point mutagenesis identified N352, R353, D382, G443, G444, and H445 as critical residues for H disaccharide binding in GII.25 P domain, while A351 determines glycan-binding specificity.

**Discussion:**

Our findings demonstrate that GII.23/24/25 exhibit glycan-binding patterns similar to most other GII HuNoV genotypes. The structural insights provide a better understanding of virus-host evolution and inform the development of therapeutic strategies against HuNoVs.

## 1 Introduction

Human noroviruses (HuNoVs) are the leading cause of viral acute gastroenteritis across all age groups (Ahmed et al., 2014). Norovirus (NoVs) are members of the *Caliciviridae* family and are currently divided into ten genogroups (GI-GX), among which GI, GII, GIII, GIV, and GIX have been detected in humans (Chhabra et al., 2019). Indeed, over 90% of HuNoV infections are attributed to GII strains, which include two tentative genotypes and 26 confirmed genotypes (GII.1-GII.27, excluding GII.15) (Chhabra et al., 2019). There are currently no commercialized vaccines or antiviral drugs.

NoVs possess positive-strand RNA genomes approximately 7.5 kb, containing three open reading frames (ORFs). ORF2 encodes the major structural protein (VP1), which is further divided into a shell (S) domain and a protruding (P) domain (Prasad et al., 2025). The P domain of NoVs plays a critical role in mediating virus-host interactions and viral antigenicity by recognizing diverse glycans, thereby facilitating or inhibiting viral infection (Tenge et al., 2021). Histo-blood group antigens (HBGAs) are glycans present on specific cell surfaces as components of glycoproteins and glycolipids, as well as in free form in saliva and other bodily secretions. These molecules determine ABO and Lewis blood group systems and function as attachment factors that influence susceptibility to and prevalence of NoV strains (Tan and Jiang, 2010, 2011, 2014; Peters et al., 2022). HuNoVs also recognize other oligosaccharides or small molecules. For example, GII.13/21 binds to secretory mucin core2, a speculated susceptibility factor. Breast milk lactose may act as a natural decoy receptor for GII.13/21, thereby reducing the risk of infection (Cong et al., 2019). GII.4 VA387 binds to gangliosides containing sialic acid and to other complex glycans (Wegener et al., 2017; Ayyar et al., 2025). Cholic acid, a key bile acid, specifically binds to HuNoV genotypes GII.1, GII.10, and GII.19 at a conserved pocket within the P domain; this binding stabilizes the P-domain loops, positioning the essential Asp375 side chain and thereby facilitating or enhancing HBGA binding (Kilic et al., 2019). The continuous evolution and mutation of strains make it necessary for us to further analyze the oligosaccharide-binding specificity and structural characteristics of the GII HuNoVs, especially the genotypes that emerged relatively late in the GII evolutionary phylogeny.

HuNoVs have undergone continuous evolution through mutations in viral genes, likely contributing to enhanced transmissibility and infectivity. Over the past decade, novel GII.4 norovirus variants have emerged every 2–3 years, driving increased global outbreak activity (Barclay et al., 2013). In addition, the emergence of the two variants GII.2 and GII.17 has been particularly noteworthy. The structural changes in their capsid proteins may enhance binding affinity to a broader range of HBGAs, which is likely closely associated with the significant increase in infections across various regions globally (Chan et al., 2015; Ao et al., 2017; Ao et al., 2018; Qian et al., 2019). Thus, the investigation of HuNoVs-glycan binding patterns and molecular mechanisms offers great potential for predicting and early warning of the emergence of rare-genotype outbreak strains.

In the present study, we elucidated the functional characteristics and structural basis underlying the recognition of glycans by the GII.23/24/25 P domains. Furthermore, through sequence alignment and structural superposition analyses, the conservation of the binding pockets within these P domains was assessed across the GII genogroup of NoVs. These findings provide insights into viral pathogenesis and may facilitate the development of targeted therapeutic strategies against this pathogen.

## 2 Materials and methods

### 2.1 Protein expression and purification

The genes encoding the P domains of GII.23 Quininde1906 (GenBank: KR232647), GII.24 Loreto6424 (MG495081), and GII.25 Dhaka1928 (MG495083) were synthesized by Genewiz (Suzhou, China). A cysteine-containing short peptide (CDCRGDCFC) was fused to the C-terminus of the P domain to stabilize P-protein formation, while the wild-type P domain sequence was used to generate P dimers (Holroyd et al., 2025). The P domain segments were cloned into the pGEX-6P-1 vector as previously described (Tan and Jiang, 2011). For protein expression, plasmids were transformed into *Escherichia coli* BL21(DE3) competent cells. Protein expression was induced with 0.4 mM isopropyl-β-D-thiogalactoside (Roche, 11411446001) at 22°C for 16–20 h. Cells were harvested and resuspended in phosphate-buffered saline (PBS, 140 mM NaCl, 2.7 mM KCl, 10 mM Na_2_HPO_4_, 1.8 mM KH_2_PO_4_, pH 7.4). Clarified lysates were loaded onto Glutathione Sepharose 4B GST-tagged protein purification resin (Cytiva, 17075601), and P proteins were eluted by PreScission protease digestion (Beyotime, P2302). P dimers were further purified using a Superdex 200 Increase ^10/300^GL gel-filtration column (Cytiva, 28990944). Mutations were constructed using the QuikChange site-directed mutagenesis kit (Beyotime, D0206S).

### 2.2 Saliva binding assay

An enzyme-linked immunosorbent assay (ELISA) was performed to assess binding between saliva samples and the P proteins of norovirus as previously described (Ao et al., 2018). The 96-well microplates were coated overnight at 4°C with heat-inactivated saliva samples (diluted 1:10,000) from both ABO secretor and non-secretor individuals. After five washes with PBS-T (PBS supplemented with 0.05% Tween 20), plates were blocked with 5% skim milk in PBS for 2 h at 37°C. Subsequently, 0.5 μg/well of P proteins were added and incubated overnight at 4°C. Following five washes with PBS-T, rabbit anti-P domain polyclonal antibody (1:6,000) that was generated and validated as previously described (Cong et al., 2019) was incubated at 37°C for 2 h. After washing with PBS-T, HRP-conjugated goat anti-rabbit IgG (1:1,000, Beyotime, A0208) was incubated at 37°C for 1 h. Following extensive washing, 3,3′3llow-tetramethylbenzidine (TMB) substrate reagent (BD Biosciences, 555,214) was added to each well in the dark at room temperature. The reaction was terminated with 1 M H_3_PO_4_, and absorbance was measured at 450 nm. The cutoff for positive signal of Optical Density at 450 nm (OD_450_) was defined as the mean OD_450_ value of negative control plus three times the standard deviation (Xie et al., 2020). For data normalization, the raw OD_450_ value of sample well was subtracted by the average OD_450_ value of blank control well to account for background absorbance.

### 2.3 Glycan binding assay

A glycan binding assay was conducted as described previously (Ao et al., 2018). Briefly, 20 μg/well of purified P proteins was coated onto 96-well microplates overnight at 4°C. Following blocking with 5% non-fat milk in PBS for 2 h at room temperature, wells were incubated with 0.2 μg/well of the following biotinylated glycans overnight at 4°C: A trisaccharide (A), B trisaccharide (B), H disaccharide (H), H type 1 (H1), H type 2 (H2), H type 3 (H3), Lewis a (Le*^a^*), Lewis x (Le*^x^*), Lewis b (Le*^b^*), Lewis y (Le*^y^*), type I precursor (Lec), and type II precursor (LacNAc) (GlycoTech). After five washes with PBS-T, HRP-conjugated streptavidin (1:1,500, Proteintech, SA00001-0) was added and incubated for 1 h at 37°C. After an additional wash, TMB substrate reagent was added for color development. The reaction was terminated with 1 M H_3_PO_4_ and absorbance was measured at 450 nm. The cutoff for the positive signal of Optical Density at 450 nm (OD_450_) was defined as the mean OD_450_ value of the negative control plus three times the standard deviation (Xie et al., 2020). For data normalization, the raw OD_450_ value of sample well was subtracted by the average OD_450_ value of blank control well to account for background absorbance.

### 2.4 Protein crystallization

The purified P dimers of norovirus strains GII.23 Quininde1906, GII.24 Loreto6424, and GII.25 Dhaka1928 were concentrated to 5–10 mg/mL for crystallization. Initial screening utilized commercial kits at 18°C using the sitting-drop vapor diffusion method with a drop ratio of 1 μL protein solution to 1 μL reservoir solution. Crystallization conditions were as follows: GII.23 in 10% (v/v) PEG 200, 0.1 M BIS-TRIS propane (pH 9.0), and 18% (w/v) PEG 8,000; GII.24 in 0.1 M sodium acetate trihydrate (pH 4.0) with 10% (w/v) PEG 4,000; GII.25 (native and H-dissaccharide complex) in 0.17 M ammonium sulfate, 0.085 M sodium cacodylate trihydrate (pH 6.5), 25.5% (w/v) PEG 8,000, and 15% (v/v) glycerol, with complex formation involving pre-incubation with H disaccharide (Dextra Laboratories) at 1:50 molar ratio for 5 h at 4°C. For X-ray diffraction, crystals were flash-cooled in liquid nitrogen using cryoprotectant solution (reservoir supplemented with 20% glycerol).

### 2.5 Data collection, processing and analysis

X-ray diffraction data were collected at Shanghai Synchrotron Radiation Facility (SSRF) BL19U1 and processed using HKL2000 (version 712) (Xiao et al., 2024). Data were further processed and scaled using CCP4 software (version 9) (Winn et al., 2011). The P domain structure was determined by molecular replacement using PHASER, with the crystal structure of GII.12 P domain (PDB: 3R6J) as a search model. The model was further refined using Phenix.refine in PHENIX (Adams et al., 2010; Afonine et al., 2012). Data collection and refinement statistics were summarized in Table 1. Amino acid sequence alignment was conducted using ClustalX software (version 2.1), and the result was visualized using the online ESPript software (Gouet et al., 1999; Larkin et al., 2007). Molecular docking of GII.25 P domain with B trisaccharide was performed using the CB-Dock2 server (Liu et al., 2022). Structural superposition and visualization were performed using PyMOL software (version 2.6.2) (Delano, 2002). Visualization of protein-oligosaccharide interactions was performed using LigPlot^+^ software (version 2.3) (Laskowski and Swindells, 2011).

**TABLE 1:** Crystallographic X-ray diffraction and refinement statistics.

Parameter^a^	GII.23	GII.24	GII.25	GII.25-H
**PDB code**	22ZV	22ZW	22WZ	22XH
**Data collection**				
Space group	P212121	P212121	C121	C121
**Cell dimensions**				
*a*, *b*, *c* (Å)	60.832 107.313 220.661	73.629 80.304 134.287	108.839 53.491 109.425	108.950 53.392 109.355
α, β, γ (°)	90, 90, 90	90, 90, 90	90, 102.699, 90	90, 102.897, 90
Resolution (Å)	50.00–2.00 (2.03–2.00)	50.00–2.35 (2.43–2.35)	50.00–1.50 (1.55–1.50)	50.00–1.60 (1.66–1.60)
*R*_merge_ (%)^b^	0.042 (0.698)	0.054 (0.866)	0.076 (0.703)	0.096 (0.679)
*I/*σ*I*	13.4 (3.1)	9.9 (2.4)	22.716 (2.955)	16.054 (2.454)
Completeness (%)	100 (100)	99.7 (100)	97.87 (83.66)	98.54 (88.92)
Redundancy	9.8 (10.4)	10 (10)	6.6 (6.5)	5.5 (5.5)
**Refinement**				
Resolution (Å)	47.76–2.00	38.28–2.35	45.31–1.50	47.7–1.60
No. reflections	93,530	33,685	98,098	80,633
*R*_work_/*R*_free_	0.1824/ 0.2280	0.2383/ 0.2786	0.1837/ 0.2035	0.1801/ 0.1966
**No. atoms**				
Protein	10,606	5,085	4,847	4,836
Ligand/ion	0	2	0	44
Water	838	214	766	683
***B*-factors**				
Protein	28.5	42.5	18.26	18.83
Water	34.0	33.8	28.12	27.66
Ligand/ion	–	–	–	39.94
**R.m.s. deviations**				
Bond lengths (Å)	0.007	0.011	0.003	0.003
Bond angles (°)	1.397	1.49	0.618	0.646
**Ramachandran plot**				
Favored (%)	94.88	95.14	98.03	98.03
Allowed (%)	4.03	4.86	1.97	1.97
Disallowed (%)	0.09	0.00	0.00	0.00

^a^Values in parentheses are given for the highest resolution shell. ^b^Rmerge = Σhkl |I- < I > |/ΣhklI, where I is the intensity of unique relfection hkl and < I > is the average over symmetry-related observations of unique reflection hkl, hkl is the reflection indices.

## 3 Results

### 3.1 Glycan binding specificity of GII.23/24/25 P domains

**FIGURE 1:**
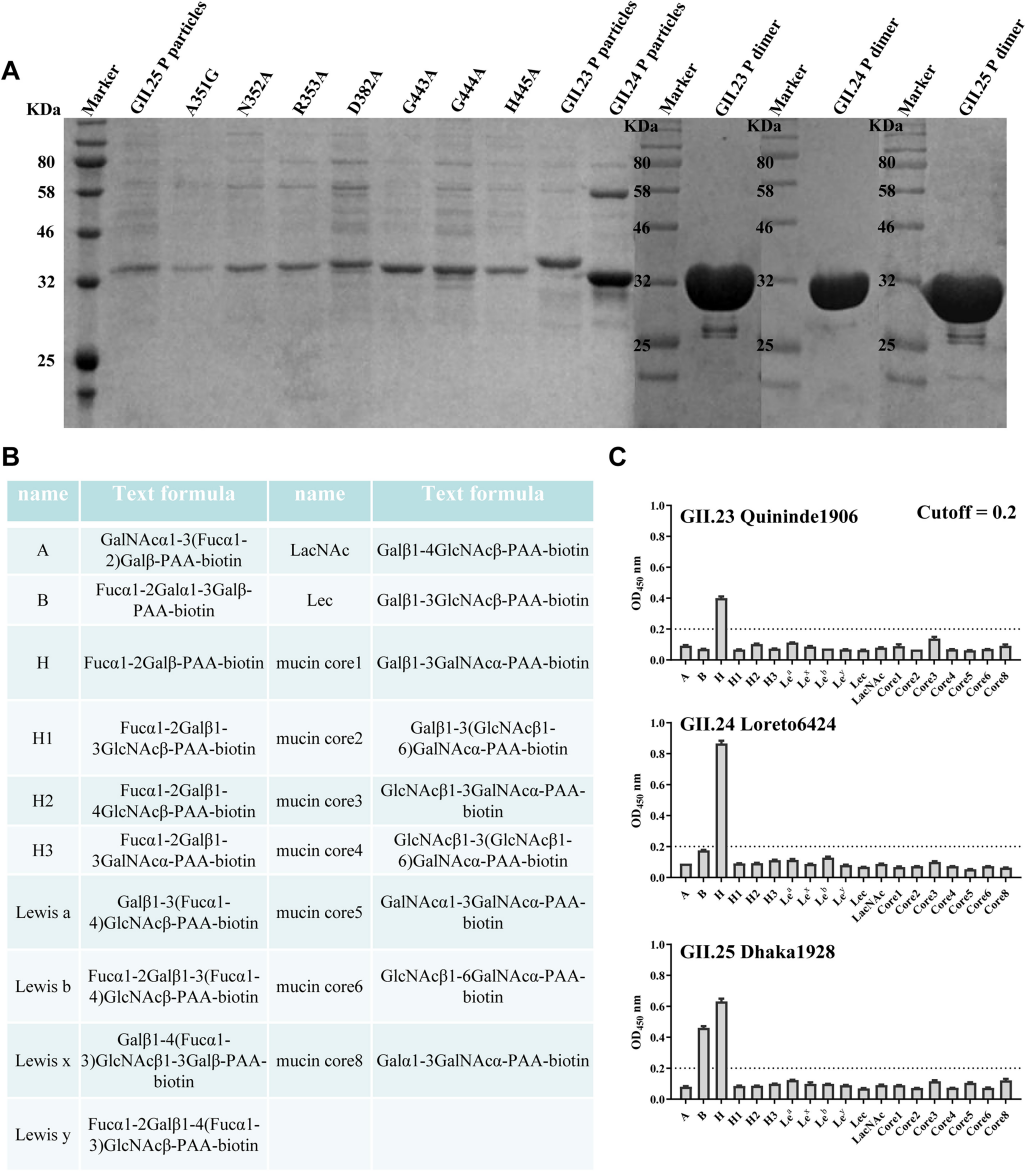
Glycan binding specificity of the GII.23/24/25 P domains to HBGAs and mucin core families. **(A)** SDS-PAGE analysis of three GII.23/24/25 P proteins, seven GII.25 mutant P proteins, and three GII.23/24/25 P dimers. **(B)** Glycans used in this study. **(C)** The binding of GII.23/24/25 P proteins to various synthetic polyacrylamide (PAA)-biotin-conjugated glycans (*x*-axis) was tested at a concentration of 0.2 μg/well. The experiment was repeated twice independently and data are mean and standard deviation (error bars) absorbance values. OD_450nm_, optical density at 450 nm.

To identify glycan-binding profiles of norovirus strains GII.23 Quininde1906, GII.24 Loreto6424 and GII.25 Dhaka1928, recombinant P proteins were expressed as glutathione S-transferase (GST) fusion proteins and subsequently released by PreScission protease cleavage at 4°C (Figure 1A). We further conducted a glycan-binding assay using GII.23/24/25 P proteins and synthetic oligosaccharides, including types A, B, H, H1, H2, H3, Le*^a^*, Le*^x^*, Le*^b^*, Le*^y^*, Lec, LacNAc, and mucin cores 1–8, with the exception of core 7 (Figure 1B). The results indicate that GII.23/24 P proteins specifically bind to the H disaccharide, while GII.25 binds to both H disaccharide and B trisaccharide (Figure 1C).

### 3.2 Saliva binding specificity of GII.23/24/25 P domains

**FIGURE 2:**
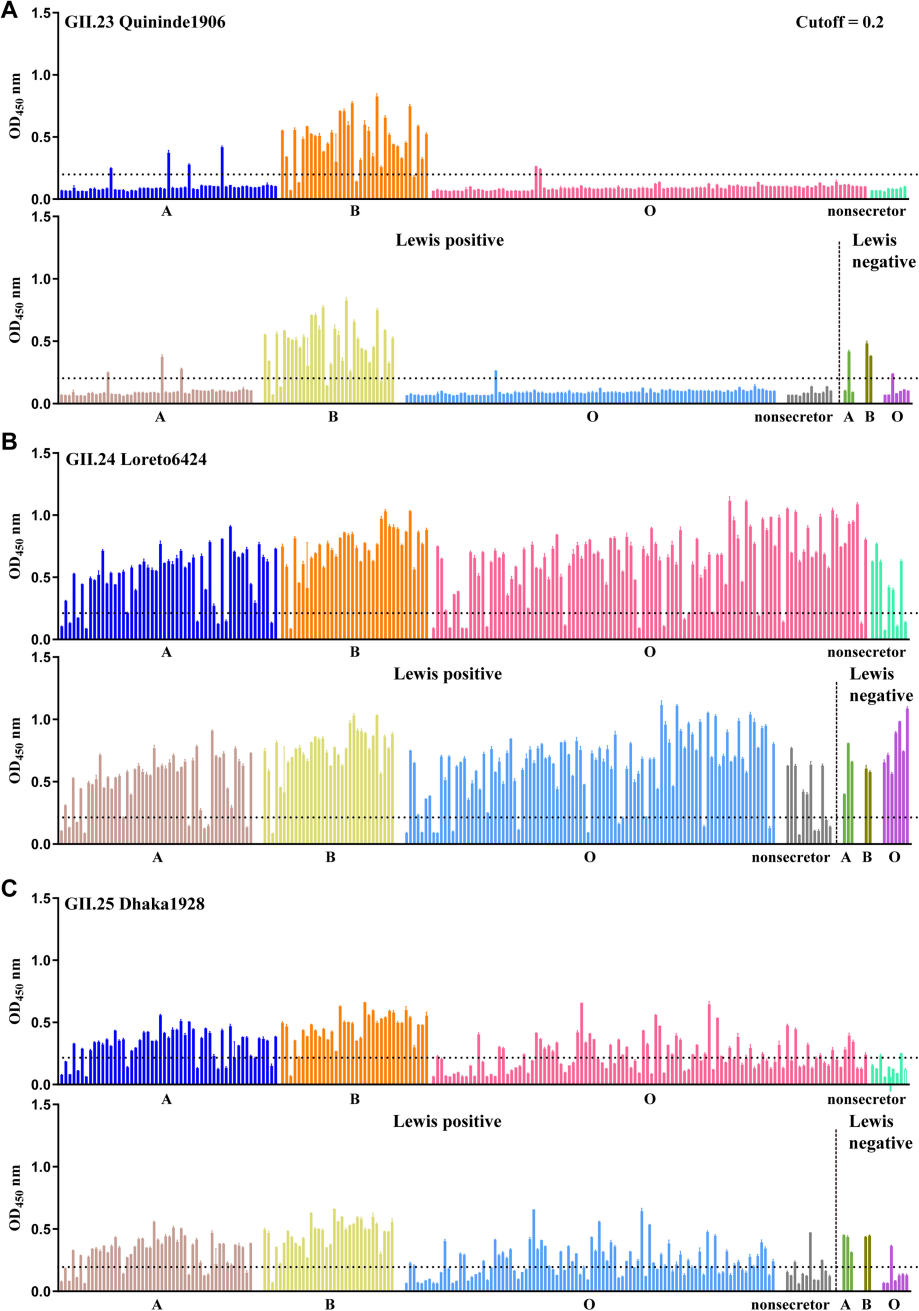
Saliva binding specificity of GII.23/24/25 P domains. Saliva-based binding assay showed the binding signals (*y*-axis) of GII.23 **(A)**, GII.24 **(B)** and GII.25 **(C)** to ABO as well as lewis type samples (*x*-axis) at 0.5 μg/well.

In the saliva binding assay, the samples collected from individuals with A, B, O blood group secretor (A, B, O) and non-secretor phenotypes were used to determine the binding signals of GII.23/24/25 P proteins to saliva. The results showed that GII.23 P protein exhibited positive binding mainly to saliva samples from B secretors, but no binding was observed in A/O secretors and non-secretors, except for a few individual samples. The Lewis classification method indicated that GII.23 mainly binds to the saliva samples of Lewis positive/negative B (Figure 2A). GII.24 P protein exhibits broad binding affinity for most saliva samples (including secreted type A, B, O, and non-secreted types), which is consistent with the result of Lewis classification method (Figure 2B). In contrast, the saliva-binding profile of GII.25 P protein is similar to that of GII.24, but its binding affinity is weaker. Specifically, GII.25 bound to B secretors, a portion of A/O secretors, with minimal binding to non-secretor samples. The Lewis classification method demonstrated that GII.25 protein exhibits binding capability to a variety of Lewis-positive and Lewis-negative samples (Figure 2C). The GST protein was used as a negative control.

### 3.3 Crystal structures of the GII.23/24/25 P dimers and GII.25 complex with H disaccharide

**FIGURE 3:**
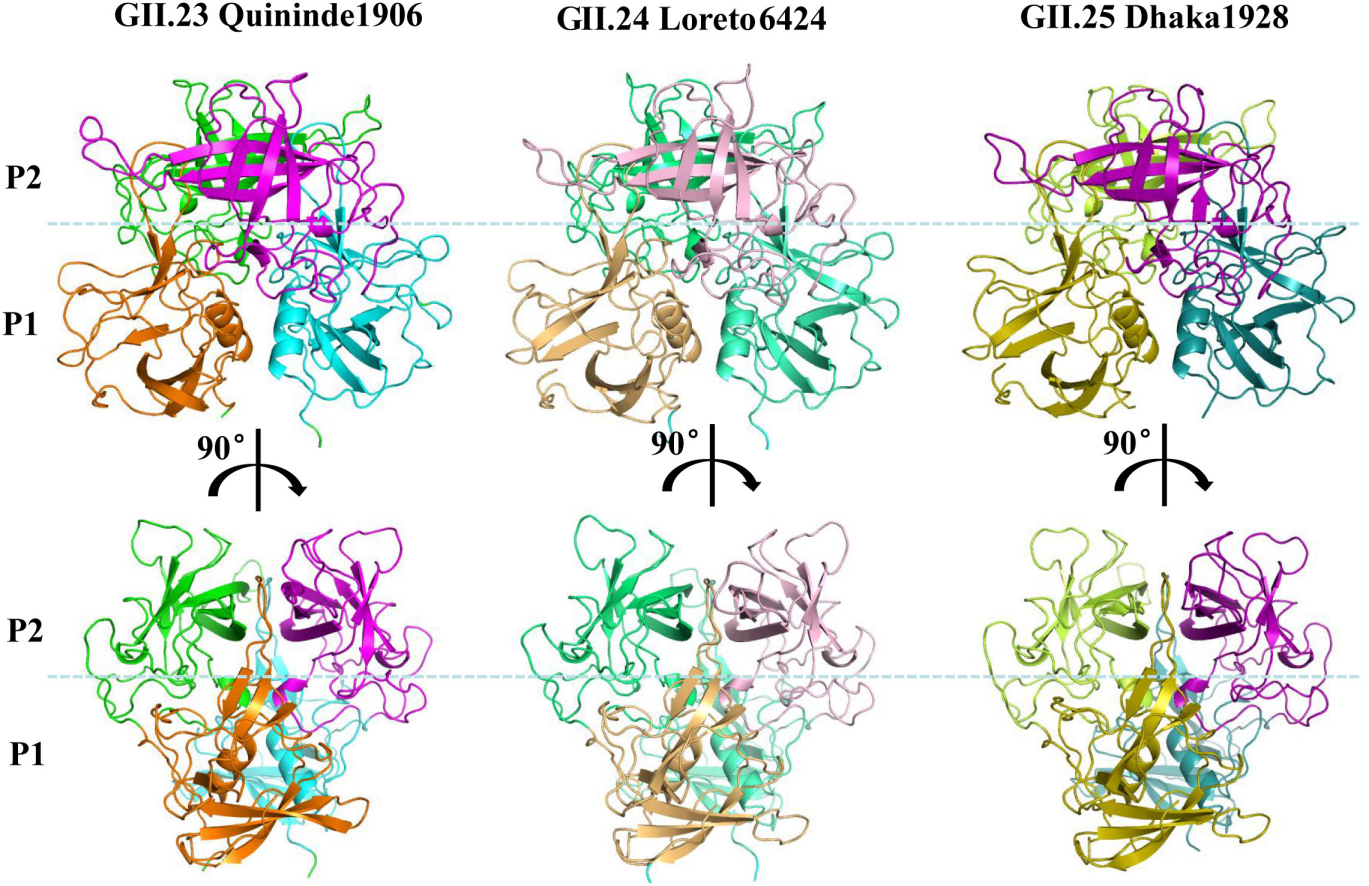
The crystal structures of the GII.23/24/25 P dimers. Structures of GII.23 two P1 and P2 proteins (cartoon model) are in orange and cyan, magentas and greens, respectively. Structures of GII.24 two P1 and P2 proteins (ribbon model) are in lightorange and greencyan, lightpink, and limegreen, respectively. Structures of GII.25 two P1 and P2 proteins (ribbon model) are in olive and deepteal, purple and limon, respectively. The dotted blue line marks the boundary between the P1 and the P2 subdomains.

**FIGURE 4:**
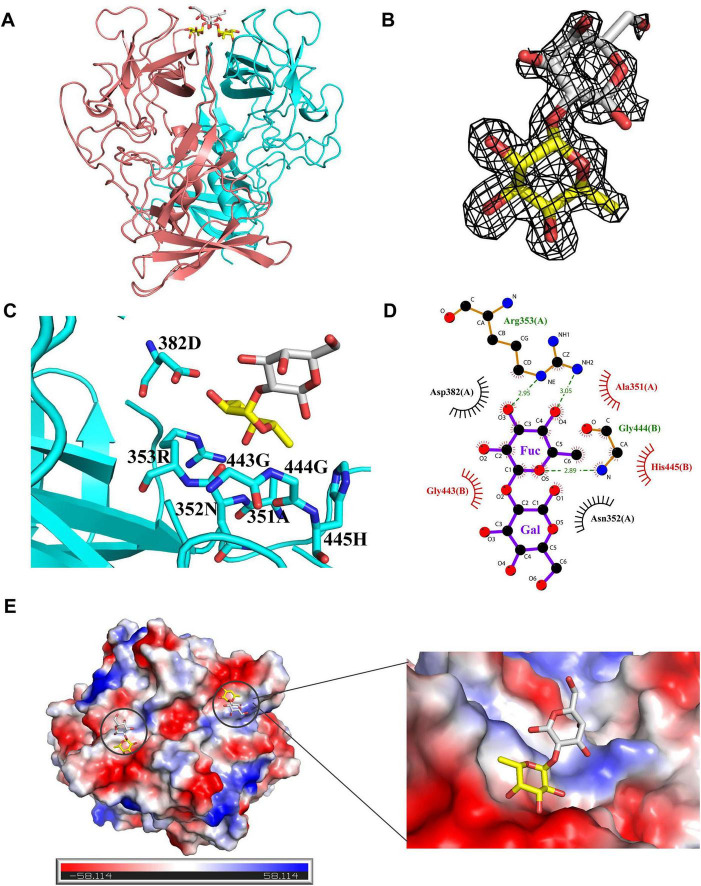
Crystal structure of the GII.25 P dimer complex with H disaccharide. **(A)** The cartoon represents the complex formed by GII.25 P dimer in complex with H disaccharide. Cyan and red, the two monomers of the GII.25 P dimer. Fucose (Fuc), yellow; Galactose (Gal), gray. **(B)** The mesh map of H disaccharide was contoured at 1 (gray) around the selection site, with a coverage of 1.6 Å radius. **(C)** The network of interactions between GII.25 and H disaccharide. The amino acids involved in the interaction are shown as cyan sticks. Fuc, yellow; Gal, gray. **(D)** Schematic diagram of the GII.25-H disaccharide interaction. Hydrogen bonds, green dashed lines; hydrophobic contacts, red arcs; van der Waals contacts, black arcs. Black, red, and blue represent carbon, oxygen, and nitrogen atoms, respectively. **(E)** The electrostatic surface potentials of the glycan binding sites of GII.25 P dimer. GII.25 P dimer is shown in surface representation. The blue and red color indicates the positive and negative electrostatic surface potentials, respectively. The glycan binding sites are framed by the black circles. A large view of the electrostatic surface potentials of the glycan binding sites is shown on the right.

The P dimers of GII.23 Quininde1906 and GII.24 Loreto6424 crystallized in space group P2_1_2_1_2_1_, whereas GII.25 Dhaka1928 crystallized in space group C121. All three structures exhibited well-defined electron density maps, indicating high-quality diffraction data. These P dimers share typical global structures comparable to those of conventional P domains, including β-sheets, α-helices, distinct P1 and P2 subdomains, and a conserved stable fold overall (Figure 3). To characterize GII.25 P dimer interactions with H disaccharide (Fucα1-2Gal), we determined the co-crystal structure with the GII.25 P dimer with the natural ligand (H disaccharide) at 1.6 Å resolution. The disaccharide is bound at the dimer interface, with electron density visible in the (2mFo-DFc) omit map (Figures 4A,B). The fucose (Fuc) moiety is anchored within the P domain via hydrogen bonding with R353 (Chain A) and G444 (Chain B), while hydrophobic interactions involving A351 (Chain A) and G443/H445 (Chain B) (Supplementary Figure 1), together with van der Waals interactions involving D382 (Chain A), stabilize the binding pocket. In contrast, the galactose moiety only forms Van der Waals interactions with N352 (Chain A) and is oriented away from the protein surface (Figures 4C,D). The α-Fuc occupies an electronegative glycan-binding site (Figure 4E).

### 3.4 Validation of the glycans binding interface

**FIGURE 5:**
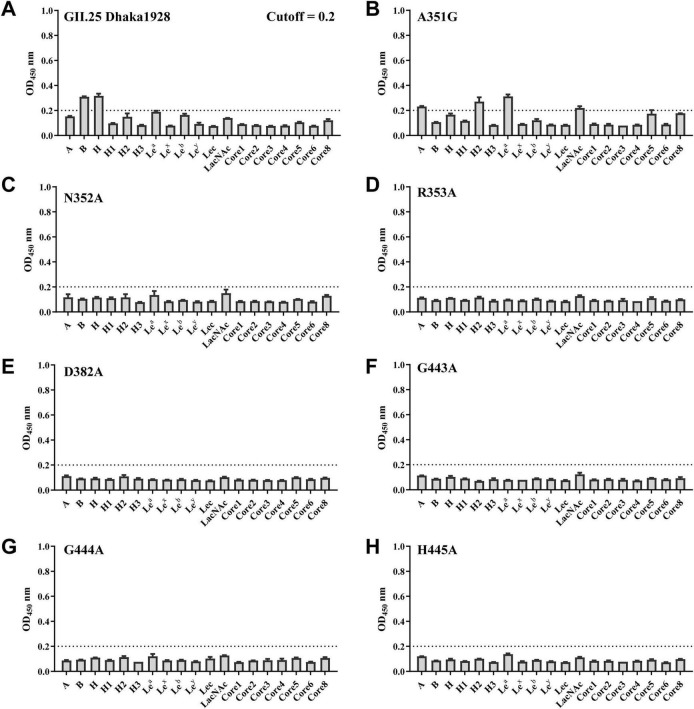
Glycan binding assay of wild type and various mutant GII.25 P proteins with a single amino acid mutation. **(A)** Glycan binding of wild type GII.25 P protein with a panel of glycans representing different HBGAs (A, B, H, H1, H2, Le*^a^*, Le*^b^*, Le*^x^*, Le*^y^*, Lec, LacNAc) and mucin core 1–6 and 8. **(B–H)** Glycan binding of seven mutant P proteins with a single amino acid mutation at the glycan-binding interface with the same panel of glycan.

**FIGURE 6:**
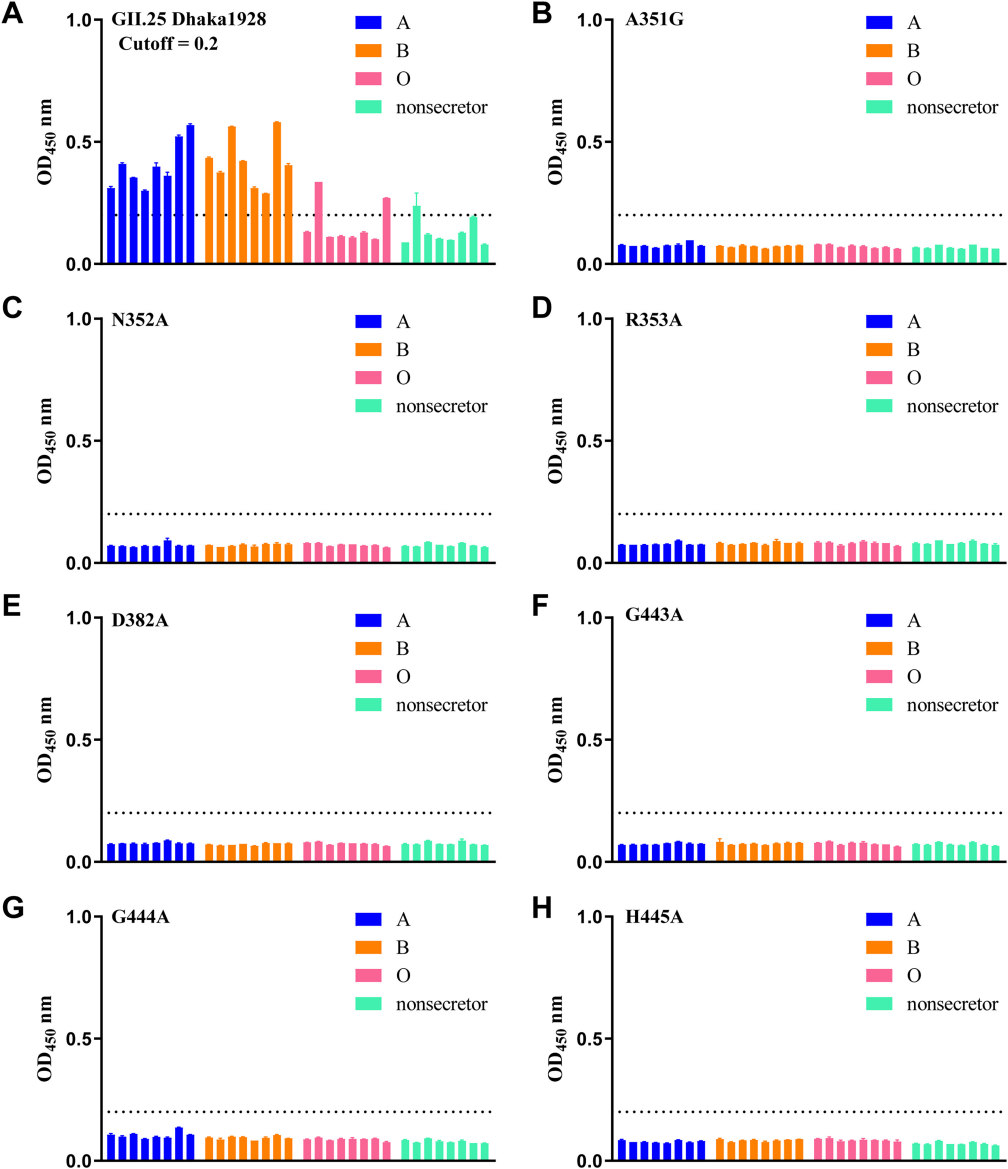
Saliva binding assay of wild type and various mutant GII.25 P proteins with a single amino acid mutation. **(A)** Saliva binding of wild type GII.25 P protein with a panel of saliva samples representing different HBGAs, secretor ABO, and non-secretor. **(B–H)** Saliva binding of seven mutant P proteins with a single amino acid mutation at the glycan-binding interface with the same panel.

To verify the functional significance of the residues involved in the GII.25-H disaccharide interaction, single-point mutants, including A351G, N352A, R353A, D382A, G443A, G444A, and H445A, were generated by site-directed mutagenesis (Figure 1A). Compared with wild-type GII.25 P protein in saliva and glycan-binding assays, all six mutants (N352A, R353A, D382A, G443A, G444A, H445A) exhibited undetectable binding to a range of saliva samples and glycan types, demonstrating that these residues at the P2 interface are essential for ligand recognition and interaction (Figures 5A,C–H, 6A,C–H). The A351G mutant not only exhibits a complete loss of saliva-binding ability, but also displays altered carbohydrate-binding characteristics. Specifically, the mutant exhibited slightly increased binding to A, H2, Le*^a^*, and LacNAc glycans, slightly decreased binding to the H disaccharide, and completely lost binding to the B trisaccharide (Figures 5B, 6B). These results indicate that residues N352, R353, D382, G443, G444, and H445 mediate GII.25-ligand interaction, while A351 modulates glycan specificity.

### 3.5 Sequence alignment and structural comparison among GII genotypes

**FIGURE 7:**
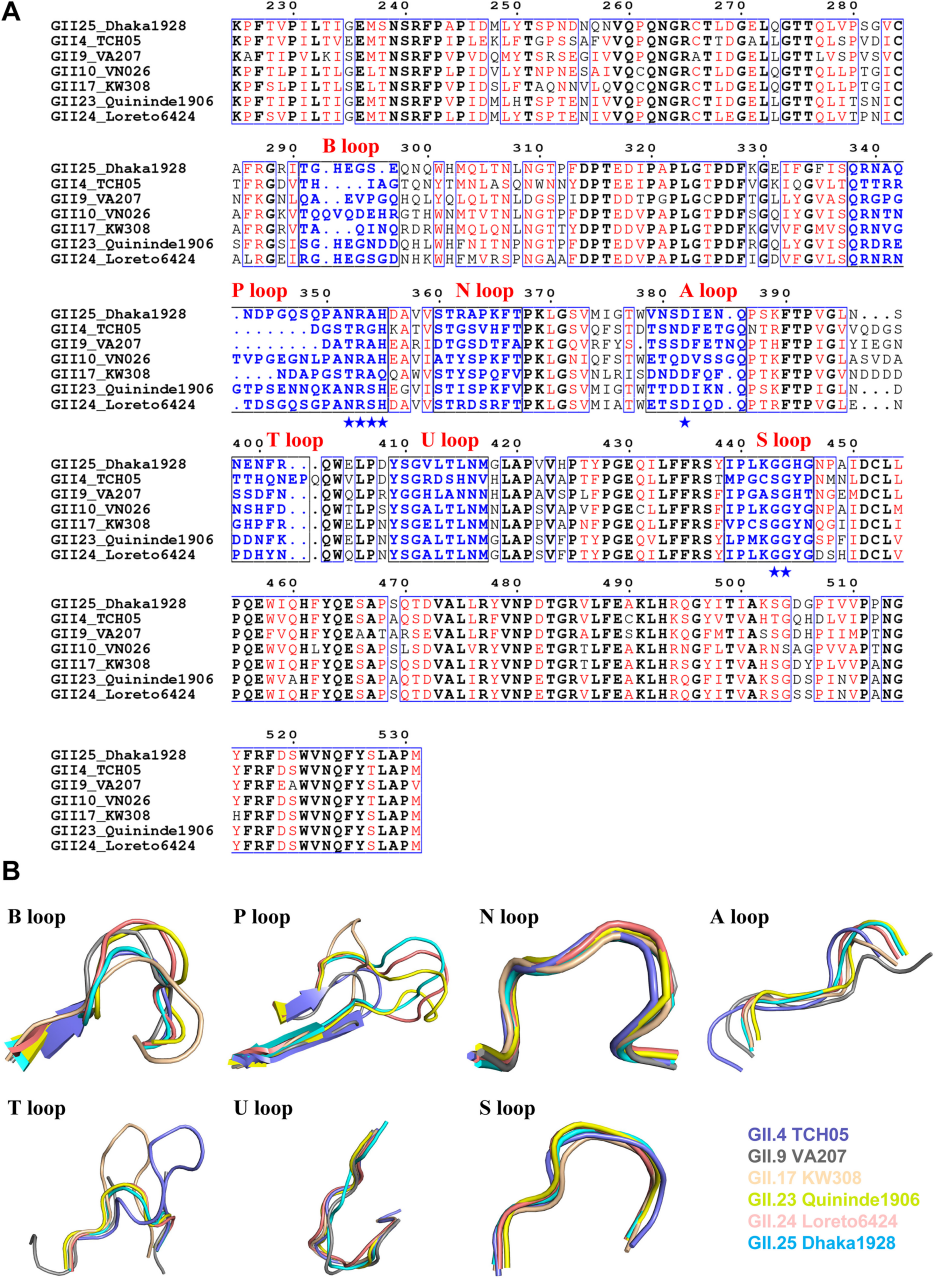
Structure-based sequence alignment of the P domains of GII.23/24/25 and different GII genotypes. **(A)** The structure-based sequence alignment of the P domains of GII.23/24/25, GII.4 TCH05, GII.9 VA207, and GII.17 KW308. Identical residues were black characters, while similar residues were shown in red characters. The conserved amino acid residues forming the conventional GII HBGAs binding interface were indicated by blue stars. The seven surface loops that constitute HBGAs binding interface were indicated by blue typeface. The letters in black indicated the identical amino acid sequences, while the red letters indicated the similar amino acids among the five NoVs. Blue boxes frame both identical and similar residues. **(B)** Comparison of the seven major surface loops of GII.23/24/25 P proteins with those of three different GII genotypes. Color schemes: salmon, GII.4 TCH05 (PDB: 3sln); gray, GII.9 VA207 (PDB: 3PUM); wheat, GII.17 KW308 (PDB: 5f4o); yellow, GII.23 Quininde1906; pink, GII.24 Loreto6424; cyan, GII.25 Dhaka1928.

Based on the VP1 sequences (Chhabra et al., 2019), phylogenetic classification of GII NoVs reveals that GII.23/24/25 cluster in the same genetic branch during viral evolution (Supplementary Figure 2). To analyze the conservation of GII.23/24/25 P domains, three well-defined strains, GII.4 TCH05, GII.9 VA207, and GII.17 KW308, were used for sequence alignment and structural superposition. Multiple sequence alignment revealed that the GII.25 P domain shares 59.1, 52.7, and 68.2% sequence identity with the P domains of GII.4 TCH05, GII.9 VA207, and GII.17 KW308, respectively (Figure 7A). The GII.25 P protein retains the conventional GII glycan-binding motifs, and its HBGA binding pocket is formed by the P-, A-, and S-loops (Supplementary Figure 4), exhibiting high structural similarity to the three aforementioned strains, with Cα root mean square deviation (RMSD) values of 0.62 Å, 0.61 Å, and 0.62 Å, respectively. The structural conformation of P domain loops is known to be critical for ligand binding (Kubota et al., 2012). The structural comparison indicates that the N-, U-, and S-loops are relatively conserved, while the A-, B-, P-, and T-loops display low sequence identity and high structural deviation (Figure 7B). Notably, GII.23/24/25 maintain conserved GII glycan-binding characteristics, suggesting the potential emergence of later-identified GII NoVs with similar receptor-binding properties.

### 3.6 Structural comparison of GII.25-H disaccharide complex with GII.23 and GII.24 P domains

**FIGURE 8:**
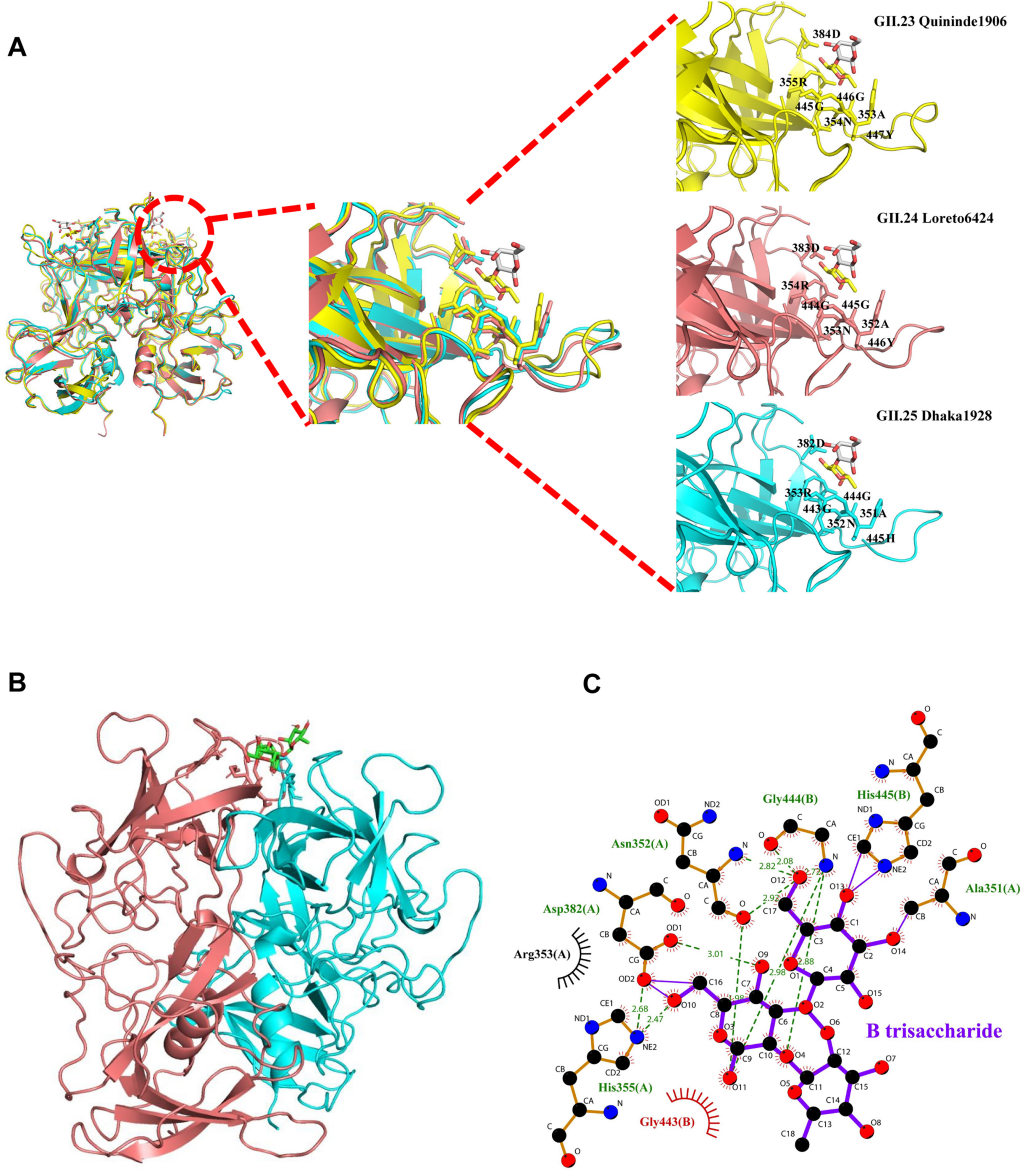
Docking structure of the interaction between the noroviruses (NoVs) P proteins and glycans. **(A)** A cartoon representation of the structure superimposition of GII.23/24 and GII.25 complex with H disaccharide. GII.23 Quininde1906, yellow; GII.24 Loreto6424, red; GII.25 Dhaka1928, cyan. The amino acids of the P proteins interacting with H disaccharide are shown in stick representation. H disaccharide, Fuc, yellow; Gal, gray. Black, red, and blue in glycans represent carbon, oxygen, and nitrogen atoms, respectively. **(B)** Molecular docking simulation for the interaction between GII.25 P protein and B trisaccharide. The dimer of GII.25 was painted in cyan and red. B trisaccharide was shown in stick representation. **(C)** Schematic diagram of the GII.25-B trisaccharide interaction. Hydrogen bonds, green dashed lines; ionic bonds, purple solid line; hydrophobic contacts, red arcs; van der Waals contacts, black arcs. Black, red, and blue represent carbon, oxygen, and nitrogen atoms, respectively.

To analyze the structural features of the interaction between GII.23/24 and H disaccharide, we superimposed the structure of the GII.25-H disaccharide complex onto that of GII.23/24. Structural alignment revealed similar folding architecture between GII.23/GII.24 and GII.25-H disaccharide complex, with Cα RMSD values of 0.69 and 0.50 Å over 492 aligned residues. Previous studies have shown that the P domain of GII.23 Loreto1847 binds to fucose through residues R355 and D384 (Holroyd et al., 2025). Similarly, the P domain of the GII.24 Loreto 1972 interacts with the fucose moiety of HBGAs via a conserved binding pocket composed of residues N353, R354, D383 and G445 (Hansman et al., 2024). These findings are consistent with our structural superposition results (Figure 8A). Structural analysis of HBGA binding interfaces of huNoVs reveals that most GII genotypes recognize HBGAs via a set of scattered and conserved amino acids (Liu et al., 2015). Sequence alignment of interaction interfaces shows that the key residues responsible for binding glycans in GII.23/24/25 are highly conserved among most GII strains (Supplementary Figure 3A). Furthermore, residues A351, R353, D382, G443, and G444, which are involved in the interaction between GII.25 and the Fuc moiety of H disaccharide, are also well conserved, whereas residue H445 exhibits lower sequence conservation (Supplementary Figure 3B). Despite the overall structural similarity, analysis revealed sequence variations and conformational deviations in glycan-binding sites at their respective H disaccharide interfaces. Although sequence alignment shows residue H445 (GII.25) at the structurally equivalent position of Y447 (GII.23) and Y446 (GII.24), this substitution did not disrupt H-disaccharide binding affinities (Figure 8A). The predicted interaction diagrams indicate that the binding of the H-disaccharide to the GII.23 P protein is mediated by hydrogen bonds involving R355, hydrophobic interactions with A353/G445/G446/Y447, and van der Waals forces involving N354 and D384 (Supplementary Figure 5A), whereas binding to the GII.24 P protein is driven by hydrogen bonds involving R354, hydrophobic interactions with A352/G444/G445/Y446, and van der Waals forces involving N353 and D383 (Supplementary Figure 5B). Furthermore, the interaction between GII.25 P dimer and B trisaccharide was predicted using the CB-Dock2 server. The results indicated that the binding pocket is located at the interface of the dimer and is composed of residues A351, N352, R353, H355, D382, G443, G444, and H445. The B trisaccharide forms interactions with these residues through ionic bonds, hydrogen bonds, hydrophobic interactions, and van der Waals interactions (Figures 8B,C).

## 4 Discussion

The continuous variation and evolution of NoVs remain the most critical challenge for controlling HuNoV infections. The majority of sporadic and outbreak cases are associated with GII NoVs, and recent research has focused on the glycan-binding characteristics and molecular structures of viral variants that transitioned from historically low prevalence to high-intensity outbreaks and those with the continuous mutation of prevalent strains. Despite the identification of later-identified GII NoVs in recent years, their glycan-binding properties remain largely uncharacterized (Chhabra et al., 2018). Here, we characterize the saliva- and glycan-binding profiles and present high-resolution crystal structures of P domains from later-identified GII.23/24/25 strains. These results provide a molecular basis for assessing the potential epidemic risk of GII.23/24/25 strains and for guiding the development of antiviral vaccines.

HuNoV variants exhibiting novel HBGA-binding properties may target new populations and potentially trigger global outbreaks of acute gastroenteritis, with oligosaccharide-based binding patterns serving as key determinants that are positively correlated with viral prevalence (Shanker et al., 2014; Chan et al., 2015; Liu et al., 2015). The GII.4 variants exemplifies this mechanism through its broadened binding affinity for multiple oligosaccharides, including H1, H3, Le*^b^*, and Le*^y^* (Liang et al., 2021). In this study, the P proteins of GII.23 and GII.24 bound exclusively to H disaccharide, whereas those of GII.25 bound to both H disaccharide and B trisaccharide. Compared with the prevalent strain GII.4, the narrow oligosaccharide-binding spectrum likely explains the limited capacity of these strains to cause epidemic outbreaks. It is noteworthy that the results obtained from glycan and saliva binding assays for GII.23/24/25 are not fully consistent, which may be related to the components and complexity of the binding targets. Specifically, the glycan-binding assays employed synthetically derived, well-defined single glycan chains that contain only the essential oligosaccharide moieties required for viral binding. In contrast, saliva samples contain not only core functional receptors such as HBGAs but also a variety of glycoconjugates, including glycoproteins and glycolipids, which may interfere with or modulate binding specificity (Uusi-Kerttula et al., 2014; Wegener et al., 2017). Therefore, future glycomics studies should focus on identifying specific salivary ligands that mediate the fucose-dependent binding mechanism of GII.23/24/25.

The binding characteristics of the HuNoV P domains have been elucidated for most GII NoVs and HBGAs, revealing two distinct binding patterns: The unique genetic branch GII.13/21 and the conventional GII genotype. Specifically, the HBGA-binding conformations of GII.13/21 are located on the surface of each P monomer with galactose as the major binding site, whereas those of the conventional GII strains are located at the interface between the two P monomers utilizing Fuc as the dominant binding site (Tan and Jiang, 2014; Cong et al., 2019; Qian et al., 2019). In the crystal structure of GII.25-H disaccharide complex, GII.25 utilizes Fuc as the primary binding site for interaction with the H disaccharide, and the binding site is located at the interface of two P dimers with the spatial conformation of conventional structural. Meanwhile, the superimposition of the GII.25-H disaccharide complex with GII.23/24 reveals highly similar spatial conformations, indicating that GII.23/24/25 interacts with the H disaccharide via the conventional GII binding pattern. Although the sequence, surface structure, and conformations of the GII.25 P dimer are slightly different from those of the other GII strains, GII.25 still retains the ability to bind HBGAs. These results suggest that the binding characteristics of future novel genotypes evolved from GII genotypes may resemble those of conventional GII NoVs.

HuNoVs typically recognize and bind to HBGAs through the binding pocket of the P protein. The conserved central binding pocket (CBP) interacts with a common major binding saccharide (MaBS) of HBGAs, reflecting a strong selection of NoVs by the host. In contrast, the variable surrounding region engages with additional minor binding saccharides (MiBSs), explaining the different recognition patterns of the host for the norovirus (Tan and Jiang, 2014). Mutation analysis of the GII.25 P domain revealed an obvious alteration in the glycan-binding profile of the A351G mutant, characterized by enhanced binding affinity toward A, H2, Le*^a^*, and LacNAc, and reduced binding capacity for H disaccharide and B trisaccharide, indicating that residue A351 of the GII.25 P protein plays a critical role in the recognition and binding of diverse oligosaccharides. Although structural superposition reveals a residue difference between Y447/446 in GII.23/24 and H445 in GII.25, both variants are capable of binding the H disaccharide. The crystal structure of GII.25-H disaccharide complex revealed that residue H445 interacts with the Fuc moiety via hydrophobic interactions. The H disaccharide is formed through the glycosidic linkage of L-fucose and D-galactose, with specific regions exhibiting hydrophobic character (Usov et al., 2022). Tyrosine (Y446/447) possesses a large hydrophobic aromatic ring structure, enabling it to engage in hydrophobic interactions with the hydrophobic region of H disaccharide, thereby stabilizing the binding between GII.23/24 and H disaccharide. In contrast, alanine has a small aliphatic side chain with limited hydrophobic capacity, which impairs its ability to form stable hydrophobic interactions, thereby reducing the binding affinity of the GII.25 H445A mutant for H disaccharide. The predicted structure of the GII.25-B trisaccharide complex, as well as the binding assays of GII.25 H445A mutant, indicate that residue H445 plays a critical role in the recognition and binding of the GII.25 P protein to B trisaccharide, which may be related to the failure of GII.23/24 to bind B trisaccharide.

## 5 Conclusion

In summary, we have characterized the binding specificity and structural basis of GII.23/24/25 P domains, determined the conservation of the spatial conformation in their interactions with H disaccharide, and confirmed that these genotypes recognize glycans through the traditional GII binding pattern. These findings enhance our understanding of NoVs-host interactions, viral evolution, and epidemiology, thereby contributing to the development of strategies for the control and prevention of HuNoVs.

## Data Availability

The atomic coordinates and structure factors of the GII.23, GII.24, GII.25 structures, as well as the complex structure of GII.25 bound to H disaccharide, have been deposited in the Protein Data Bank (PDB), a stable and community-supported public repository for structural biology data, under the accession codes 22ZV, 22ZW, 22WZ and 22XH, respectively. The datasets will be made publicly available four weeks after deposition via the official PDB website at https://www.rcsb.org/. All other supporting data relevant to this study are provided in Table 1.
