# Supplementary material for: GII.23/24/25 noroviruses recognize glycans via a conventional glycan-binding site
Source: Front Microbiol. 2026 Mar 3;17:1767002. doi: 10.3389/fmicb.2026.1767002 (PMC12994431; doi:10.3389/fmicb.2026.1767002)
Supplement: Supplementary file 1 [file Data_Sheet_1.doc]

Supplementary Information for

**GII.23/24/25 noroviruses recognize glycans via a conventional glycan-binding site**

Hanbo Li *et al*.

Corresponding author:

[ccong_hz@163.com](mailto:ccong_hz@163.com)

zhaojund@126.com

**This PDF file includes:**

Supplementary Figures 1 to 5

**Supplementary Figures**

**
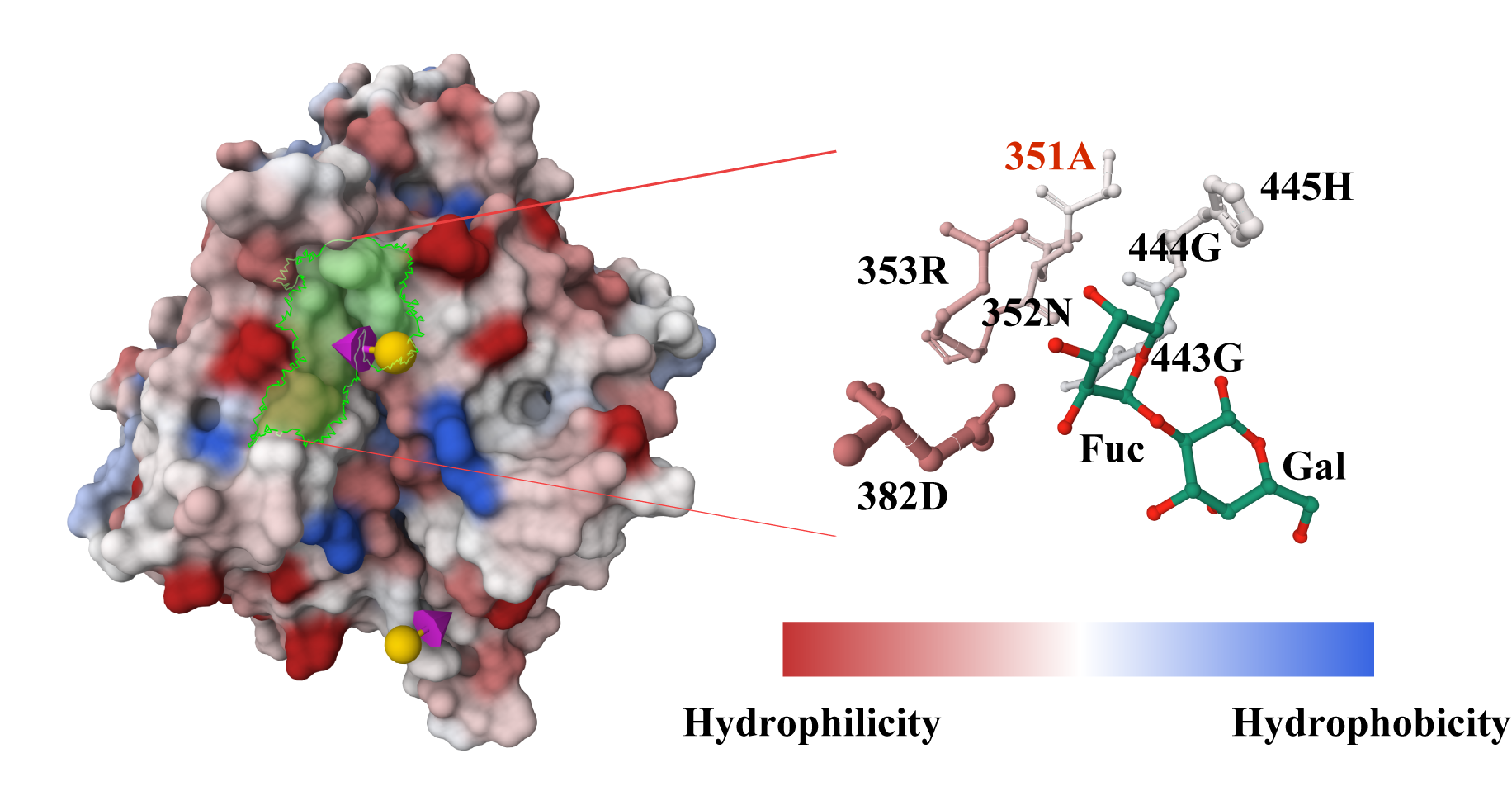
**

**Supplementary Figure 1.** Hydrophobicity distribution of the 25-H complex was plotted by Mol* Viewer tool. The left panel displays the complex surface, colored by hydrophobicity: blue indicates hydrophobic regions and red indicates hydrophilic regions. The color gradient bar denotes the polarity range. The green dashed area marks the key binding region, with purple triangles and yellow circles representing Fuc and Gal of the ligand, respectively. The right panel details the binding-site interactions, labeling residues 351A, 352N, 353R, 382D, 443G, 444G, and 445H, along with glycan components Fuc (fucose) and Gal (galactose).

**
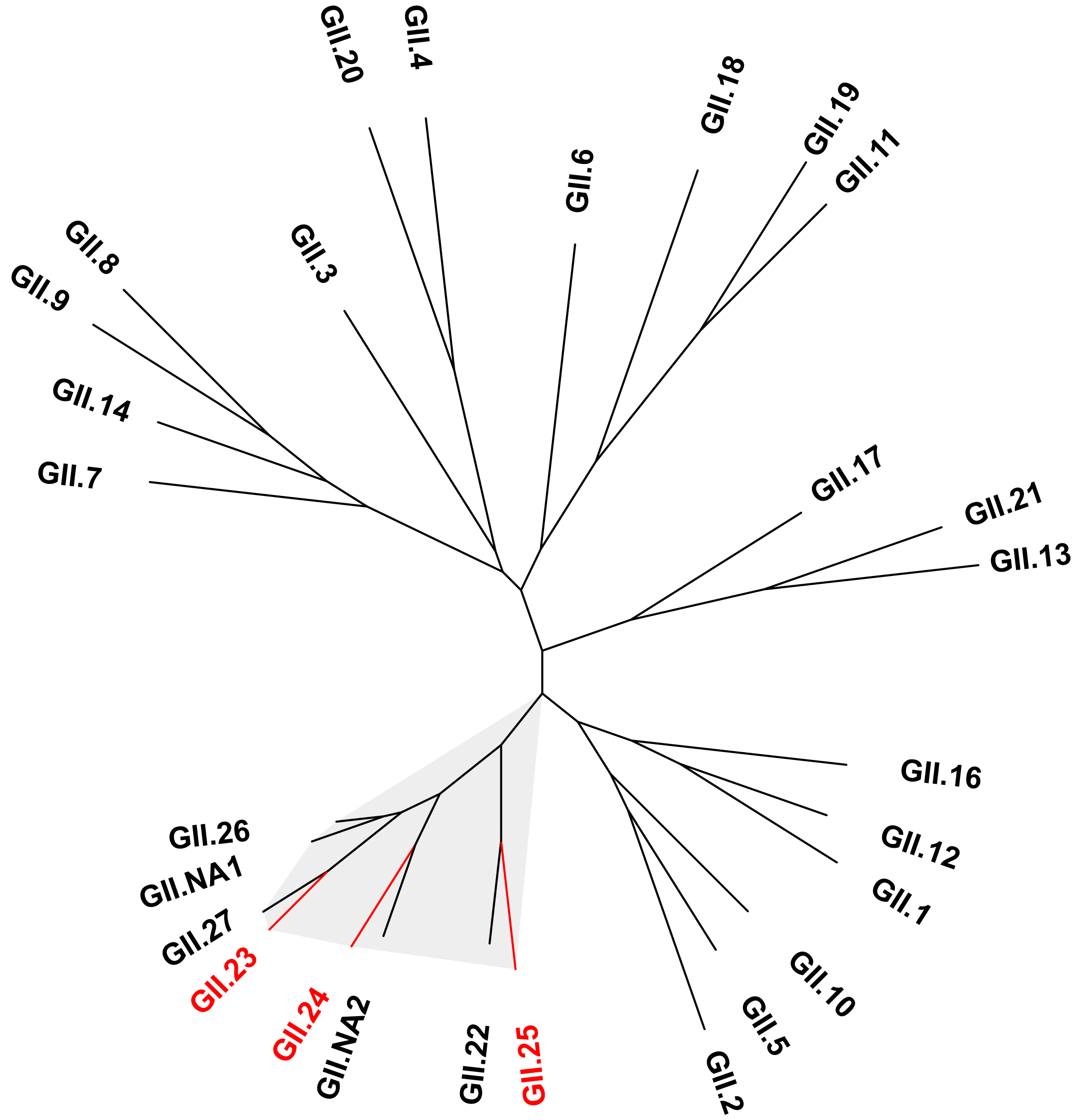
**

**Supplementary Figure 2.** Phylogenetic tree of GII NoVs based on VP1 nucleotide sequences, showing 26 norovirus genotypes and 2 unassigned (NA) genotypes. Phylogenetic analysis was conducted using maximum likelihood (MEGA 10). The resulting tree was visualized and edited using iTOL (https://itol.embl.de/). GII.23/24/25 are labelled in red. The GenBank accession numbers of the P domain sequences are: U07611.2 (GII.1 Hawaii), X81879 (GII.2 Melksham), U02030 (GII.3 TV24), X76716.1 (GII.4 Bristol), AJ277607 (GII.5 Hillingdon), AJ277620 (GII.6 Seacrof), AJ277608.1 (GII.7 Leeds), AF195848 (GII.8 Amsterdam), AY038599 (GII.9 VA207), AF427118.1 (GII.10 Erfurt), AB074893.1 (GII.11 SW918), AJ277618.1 (GII.12 Wortley), AY113106.1(GII.13 Fayetteville), AY130761.1 (GII.14 M7), AY130762.1 (GII.15 J23), AY502010.1 (GII.16 Tiffin), AY502009.1 (GII.17 CS-E1), AY823304.1 (GII.18 SW101), AY823306.1 (GII.19 QW170), EU373815.1 (GII.20 Lucken), AY675554.1 (GII.21 OIF), AB083780.1 (GII.22YURI), KT290889.1 (GII.23 Loreto1847), KY225989.1 (GII.24 Loreto1972), MG495083.1 (GII.25 Dhaka1928), KU306738 (GII.26 Leon4509), MG495077 (GII.27 Loreto0959), MG495079 (GII.NA1 Loreto1257), MG706448 (GII.NA2 PNV06929).

**
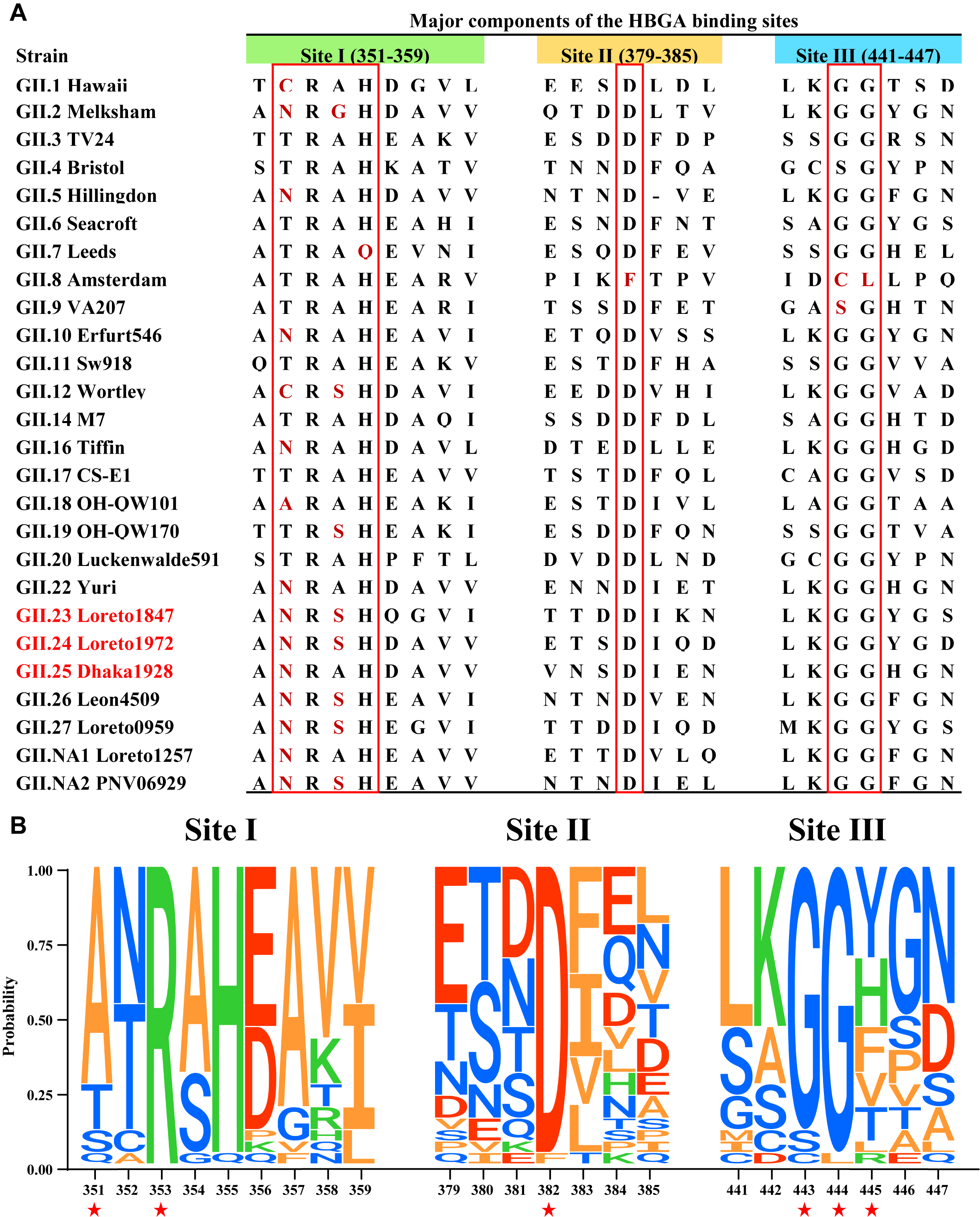
**

**Supplementary Figure 3. Sequence analysis of GII NoVs.** (**A**) P domain sequence alignment of NoVs representing each of the 26 GII genotypes with a focus on the residues at the HBGA binding interface. The conserved amino acids that form the GII conventional HBGA binding interfaces are indicated with red frames. (**B**) Sequence conservation analysis of the residue site I-III in (A). The sequence logo was plotted by OmicShare tool. Residues involved in the interaction between GII.25 and the Fuc moiety of H disaccharide are indicated by red stars.


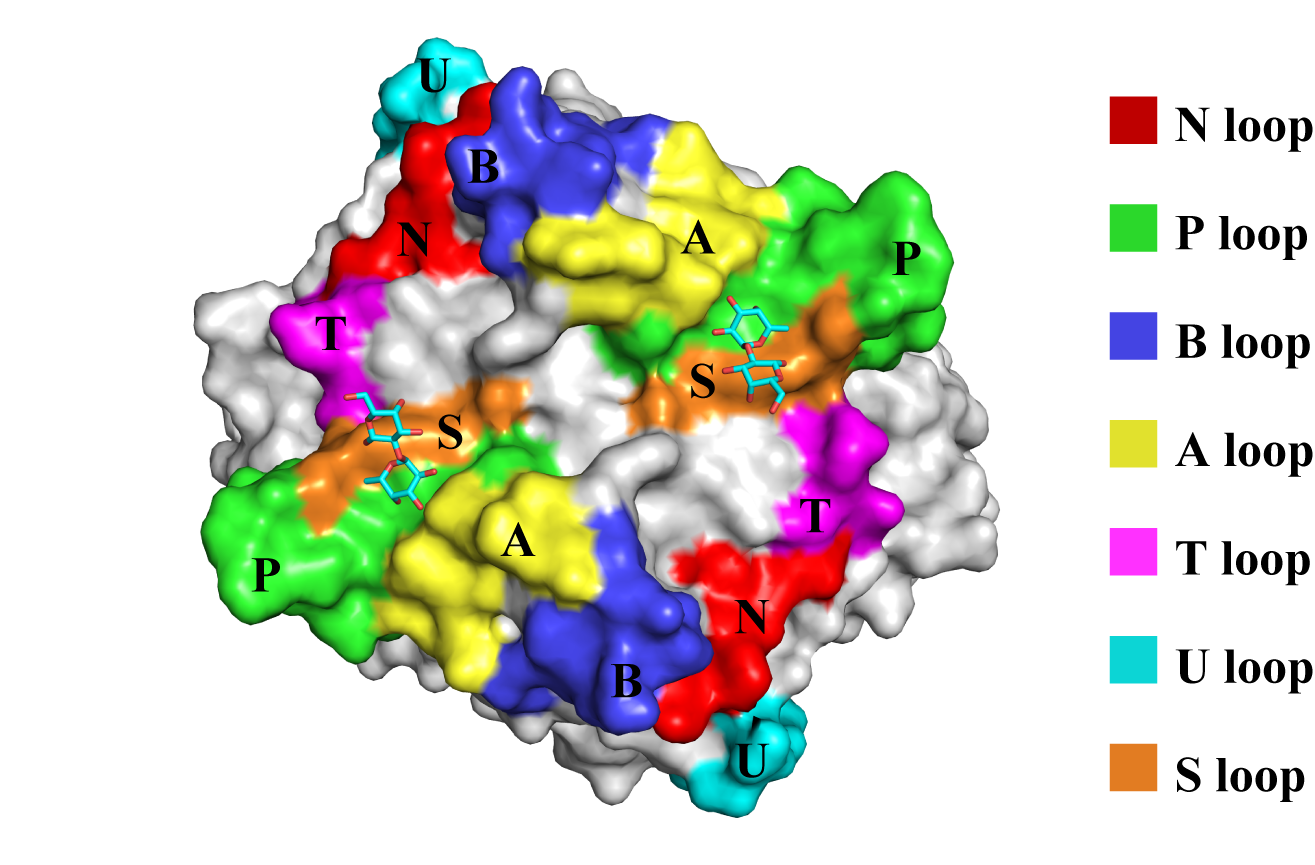


**Supplementary Figure 4.** Surface representation of the 25-H complex. The structure is displayed in surface mode. Seven distinct loops are labeled and colored as indicated in the legend: red for N loop, green for P loop, blue for B loop, yellow for A loop, magenta for T loop, cyan for U loop, orange for S loop. Glycan ligands marked by S are shown in stick representation, localized at the interface of the S loop region.


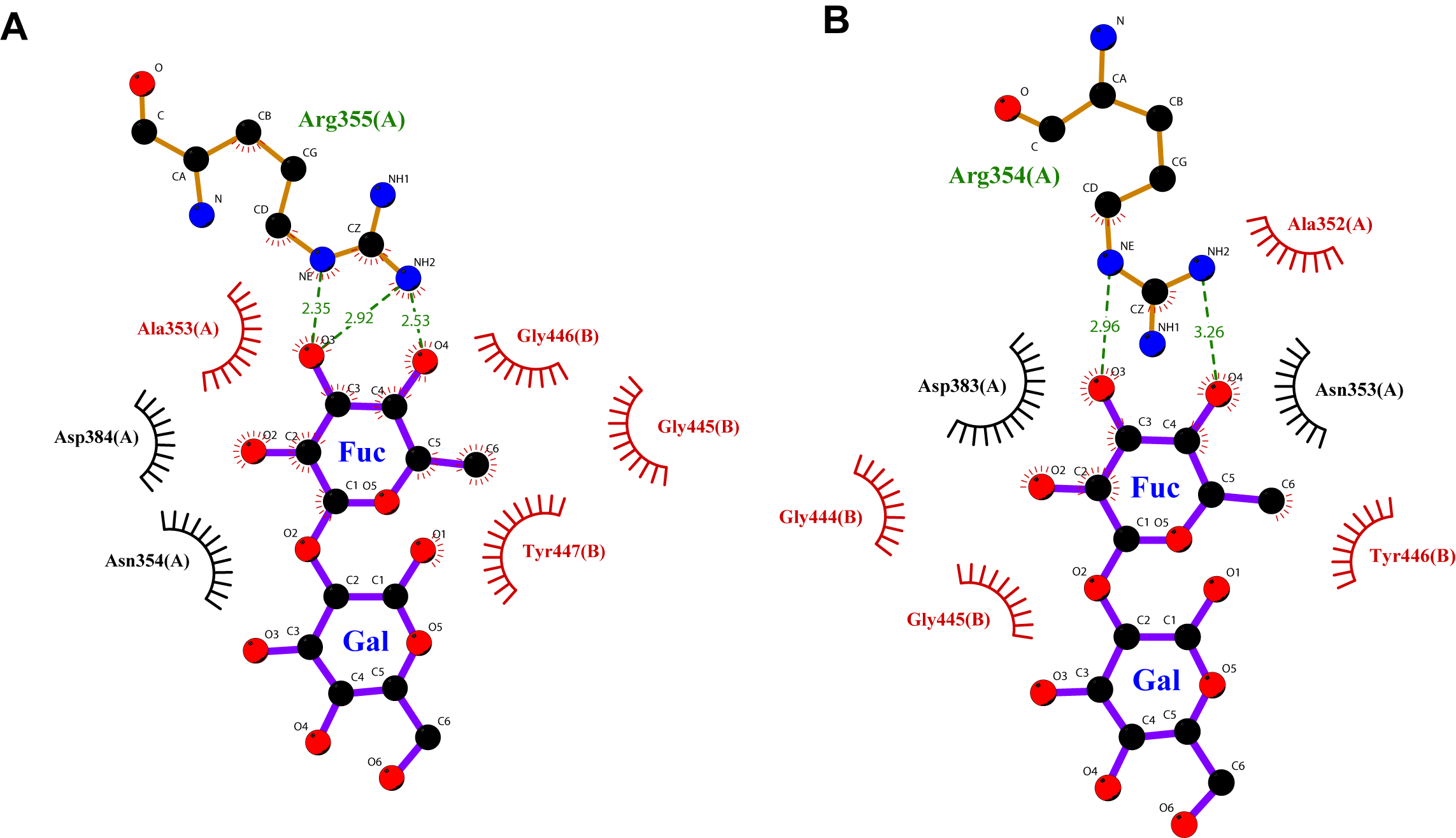


**Supplementary Figure 5.** Predicted interaction diagrams of GII.23 (A) and GII.24 (B) with H disaccharide. Green dashed lines represent hydrogen bonds; red arcs represent hydrophobic contacts; black arcs represent van der Waals contacts. Carbon, oxygen and nitrogen atoms are shown in black, red and blue, respectively.
